# Economic and Environmental Impact of Rice Blast Pathogen (*Magnaporthe oryzae*) Alleviation in the United States

**DOI:** 10.1371/journal.pone.0167295

**Published:** 2016-12-01

**Authors:** Lawton Nalley, Francis Tsiboe, Alvaro Durand-Morat, Aaron Shew, Greg Thoma

**Affiliations:** 1 Department of Agricultural Economics, University of Arkansas, Fayetteville, Arkansas, United States of America; 2 Department of Chemical Engineering, University of Arkansas, Fayetteville, Arkansas, United States of America; Fujian Agriculture and Forestry University, CHINA

## Abstract

Rice blast (*Magnaporthe oryzae*) is a key concern in combating global food insecurity given the disease is responsible for approximately 30% of rice production losses globally—the equivalent of feeding 60 million people. These losses increase the global rice price and reduce consumer welfare and food security. Rice is the staple crop for more than half the world’s population so any reduction in rice blast would have substantial beneficial effects on consumer livelihoods. In 2012, researchers in the US began analyzing the feasibility of creating blast-resistant rice through cisgenic breeding. Correspondingly, our study evaluates the changes in producer, consumer, and environmental welfare, if all the rice produced in the Mid-South of the US were blast resistant through a process like cisgenics, using both international trade and environmental assessment modeling. Our results show that US rice producers would gain 69.34 million dollars annually and increase the rice supply to feed an additional one million consumers globally by eliminating blast from production in the Mid-South. These results suggest that blast alleviation could be even more significant in increasing global food security given that the US is a small rice producer by global standards and likely experiences lower losses from blast than other rice-producing countries because of its ongoing investment in production technology and management. Furthermore, results from our detailed life cycle assessment (LCA) show that producing blast-resistant rice has lower environmental (fossil fuel depletion, ecotoxicity, carcinogenics, eutrophication, acidification, global warming potential, and ozone depletion) impacts per unit of rice than non-blast resistant rice production. Our findings suggest that any reduction in blast via breeding will have significantly positive impacts on reducing global food insecurity through increased supply, as well as decreased price and environmental impacts in production.

## Introduction

Rice is a crucial food staple for more than half of the world; accordingly, its supply must double by 2050 to keep up with food demand from population growth [1]. One of the largest impediments to increased rice production is the presence of rice blast (*Magnaporthe oryzae* fungus), which directly decreases rice yields and indirectly increases production costs [2]. Rice blast is one of the most frequent and costly rice diseases in temperate rice-growing regions worldwide [3]. To demonstrate, from 2002 to 2014, rice producers in Arkansas, Mississippi, and Louisiana planted over nine million hectares of rice to varieties that were susceptible to the rice blast fungus [4].

Yield loss from blast infections depends on varietal susceptibility, the degree of infection, and the timing of fungicide application. Some yield losses associated with blast outbreaks have reached 50% or more [5], and the cost of mitigating those blast outbreaks via fungicide application can reach over $70 ha^-1^. Although there are blast-resistant cultivars available for production in the US, most of them are hybrid lines released by RiceTec and were associated with an average seed premium of approximately $237.12 ha^-1^ in 2015, resulting in many rice producers opting for blast-susceptible varieties [6]. Correspondingly, producers tend to focus on yield potential (ceilings) of varieties instead of variability (floors), and thus may often undervalue the genetic resistance to a disease that does not raise yield potential, but raises the yield floor. In other words, blast resistance does not raise the yield potential of a given variety because blast does not occur every growing season, and yield potential is derived from a best case scenario. However, blast resistance does in fact reduce the yield variability (floor) of a variety. Consequently, this study attempts to estimate the value of raising the often overlooked “yield floor” through blast resistance.

It is both difficult and time consuming for rice breeders to breed for resistance to current strains of blast since blast is a fungus that can evolve and mutate to overcome resistance genes [7]. Blast R genes can be introduced into high yielding rice varieties through marker assisted selection. However, linkage drag can be a problem for maintaining the yield advantages [8]. The most tangible outcome of a breeding program of any type is increased yield; however, breeding for biotic and abiotic stresses (maintenance breeding) generally results in pathogen resistance, which can be viewed as mitigating potential crop losses. Economists and policy-makers tend to undervalue the opportunity cost of this type of informative agricultural research, specifically with regard to maintenance breeding. The opportunity cost of maintenance breeding can be viewed as the productivity losses that can be evaded by breeding for resistance against diseases such as rice blast. Accordingly, the substantial economic benefit that accrues from avoided yield losses through resistance to pathogens is often forgotten in the cost-benefit analysis of such breeding programs because the producers do not experience the losses, but breeding programs incur the costs to prevent them. Marasas et al. [9] estimated that the economic impact of a research program’s breeding efforts for maintenance breeding can be as great if not greater than the impact of its associated increased yields. Peng et al. [10] analyzed and emphasized the importance of maintenance breeding for rice in South Asia, where they claim a lack of genetic gain is causing a slower rate of yield increase. Their study provides strong reasoning for continuous maintenance breeding to preserve rice yield potential through improved resistance to rapidly evolving biotic stresses such as disease and insects. Similarly, Peng et al. [10] reinforce the idea that it will be difficult for global rice production to keep pace with the increasing demand for rice if maintenance breeding programs are discontinued or diminished.

Scientists have achieved blast resistance in high-yielding rice varieties using cisgenesis, a form of genetic modification [11]. Hypothetically, blast resistance could be established in all susceptible cultivars with these new breeding techniques, but this is not yet a reality due to genetically modified (GM) regulations in many rice-producing and rice-importing countries. Nonetheless, no GM rice is currently commercially available for production worldwide, even though other traditional row crops such as soybeans, cotton, and maize have GM varieties that are produced commercially around the world. Consequently, embedded disease packages for rice cultivars are not as robust as their GM crop counterparts, so disease remains a major obstacle for rice breeders and producers globally.

The legal status of cisgenic products is currently being debated among policy-makers globally [12]. The European Food Safety Authority (EFSA) has issued an opinion on the risks of new breeding techniques like cisgenesis. Andersson et al. [13] compared the biosafety of cisgenic plants with that of transgenic and conventionally bred plants, reporting that “similar hazards can be associated with cisgenic and conventionally bred plants, while novel hazards can be associated with intragenic and transgenic plants.”

The EFSA has created strict policies prohibiting the importation of GM foods [14]; thus, producers in the US have little incentive to advocate for the release of GM rice cultivars such as cisgenically produced, blast-resistant rice, which have been proven successful in field trials. As a result, the cost of blast mitigation to U.S. producers remains high; the supply of global rice has diminished; and the use of fungicides for blast control is still widespread in high income rice-growing regions of the world. Given that cisgenic breeding can greatly reduce the time from initial crossing to release, resistance genes could be delivered to producers quickly for a more comprehensive disease package that addresses blast prevention efforts. While this will not slow or mitigate the mutation of the blast fungus, it will allow for faster dissemination of technologies to combat it.

Both cisgenesis and transgenesis are plant-breeding techniques that can be used to introduce new genes into plant genomes. Distinctly, transgenesis uses genetic material from a non-plant organism, or from a donor plant that is sexually incompatible with the recipient plant; while cisgenesis involves the introduction of genetic material from a crossable, sexually compatible plant [14]. While traditional breeding can transfer blast-resistant genes like the *Pi-ta*, which was isolated in the rice variety Katy, it can take up to 10 years from initial cross to commercial release [15]. During this 10-year lag, the blast fungus can mutate and overcome the isolated resistance gene. This mutation was found in the *Pi-ta* gene case, which was isolated in 1989 and bred into multiple varieties only to be overcome by a new race of blast, *IE-1k*, which was first found in 1994 and has since caused field damage to some cultivars that were once considered blast resistant [7]. Scientists have created blast resistant Cisgenic rice via the insertion of a rice blast-resistant gene (*Pi9*) from a low-yielding wild rice variety (*Oryza minuta*) into a high-yielding and widely cultivated variety [11]. While this technology has proven successful in experimental settings [11], it has not been made commercially available in rice due to regulatory protocols put in place by many rice-importing countries.

Currently, the literature is devoid of estimations of costs and revenue effects of blast outbreaks, even though blast is responsible for approximately 30% of losses in global rice production—the equivalent of feeding 60 million people. As such, blast alleviation is a key concern in combating global food insecurity [16]. Ultimately, rice producers and consumers who experience high prices due to a diminished supply assume the costs of the absence of blast resistance in the majority of high-yielding cultivars throughout the Mid-South of the US. Therefore, this study sets out to estimate the opportunity costs of blast-resistance breeding for producers and consumers via cisgenic breeding based on (1) the area planted to blast-susceptible rice varieties from 2002–2014, (2) historical county/parish-level yield data, (3) simulated blast infection rates, (4) subsequent yield losses, and (5) fungicide applications.

Specifically, this study considers the counterfactual case of blast resistance, estimating the economic gains to producers (in the form of increased yields and decreased production costs) and consumers (as decreased rice prices), as well as the environmental benefits derived from the reduction of fungicide application. This estimation is accomplished by assuming that all rice in Arkansas, Louisiana, and Mississippi, which accounts for 73% of the acreage and 72% of the total production in the US, was sown to blast-resistant varieties from 2002–2014. It is important to present scientific, political, and consumer groups with an estimation of the economic and environmental implications of blast alleviation via cisgenics because cisgenics and its benefits could be a stepping stone in the acceptance of GM rice. While blast resistance will not be the sole determining factor for the adoption of GM rice, it provides an additional economic and environmental incentive for producers, consumers, and policy-makers in making more informed decisions about GM rice.

## Materials and Methods

In this study, the opportunity costs of blast resistance were estimated for Arkansas, Mississippi, and Louisiana using three blast outbreak and response scenarios and actual varietal planting data for 2002–2014. First, the annual varietal area planted for each rice-growing county/parish in Arkansas, Louisiana, and Mississippi was collected from 2002 to 2014 [4]. Additionally, the annual varietal yield (Mg ha^-1^) data by county/parish were collected from university-run experiment stations [17, 18, 19] and were viewed as “yield potential.” Although a gap between experimental and actual producer yields exists, Brennan [20] concluded that the most reliable sources of relative yields are cultivar trials outside of actual farm observations. While yields are often greater in experimental test plots than in producers’ fields, the relative yield difference between varieties is comparable. To explain further, not every county/parish had a university-run test plot that tested every variety sown in that county/parish. As such, all observations of unreported variety-specific yields within that state in that year were averaged, and that average yield was assigned to that county for that variety for that year. The dataset consists of 47 rice varieties, 33 rice-growing counties in Arkansas, 35 parishes in Louisiana, and 18 counties in Mississippi, with a total of 5,733 observations. Average yields by variety are reported in S1 Table.

### Blast Ratings

Blast susceptibility rankings were used for each variety and were derived from historical observations of test plots in grower fields across each state and in university-run experiment stations [17, 18, 19]. A Likert scale of blast susceptibility was used by the stations to classify rice cultivars as Resistant (R), Moderately Resistant (MR), Moderately Susceptible (MS), Susceptible (S), and Very Susceptible (VS). In Arkansas, the rankings were based on conditions that favored severe blast proliferation including excessive nitrogen rates, or low flood depth. Correspondingly, in instances where a variety became less resistant to blast (the pathogen constantly evolves to overcome the crop’s resistance), the updated rating was used. A list of blast susceptibility rankings by variety as of 2015 can be found in S1 Table. In terms of this study, the decisive factor is whether a variety is or is not blast resistant. If a variety is blast resistant, it is assumed to have neither mitigation costs, nor yield loss.

### Blast Outbreak and Yield Loss Rate

It is uncommon practice for university extension services to record blast outbreaks, or yield losses with associated blast outbreaks, with the exception of anomalies like in 2012 in Louisiana, which was classified as the worst blast outbreak in 30 years [21]. Extensive, systematic field-level yield and quality-loss estimates due to rice blast have not been developed in the US. Instead, estimates are typically recorded for corresponding crop loss depending on the inoculum density, pathogen aggressiveness, environmental conditions, cultivar susceptibility, and interaction with other cultural parameters [22]. Similarly, field-level estimates of blast loss have also been difficult to estimate because of (1) the lack of data on the numerous and often simultaneous diseases affecting rice, (2) the underground damage associated with root diseases, and (3) the lack of qualitative information on distribution and severity in commercial fields.

Outbreak acreage and percentages are scarce in the literature for Mid-South rice production, but Norman and Moldenhauer [23] provide estimates of the annual percentage of sampled commercial rice fields across Arkansas that require a fungicide application to mitigate blast outbreaks. Similar studies for Louisiana and Mississippi did not exist at the time of this study and as such, it was assumed that there were proportional fungicide applications in all three states. Ideally, state-specific distributions would become available since Louisiana typically has a higher incidence of blast than Arkansas and Mississippi, the higher incidence occurs mainly because of climatic differences between states, Louisiana has a lower probability of a hard overwinter freeze to kill the fungus. Accordingly, based on observed, historical Arkansas data, it was assumed that the mean infection rate of all susceptible varieties was 21.52% (assuming a normal distribution truncated at both 0.00% and 47.00%, the observed high). It is important to note that infection does not imply yield loss; it only implies the plant has been infected and subsequently requires fungicide to avoid potential yield loss.

Similar to the outbreak percentages, the literature is also scarce regarding replicated trials that document yield loss associated with blast on commonly cultivated rice varieties in the US. To illustrate, there are several studies that analyze only one variety in a field setting [24, 25, 26], and several more that analyze multiple varieties of varying resistance in replicated field trials [27, 22, 28, 29]. In such studies, yield losses range from 6% [23] on a moderately resistant variety (Caffey) in Louisiana, to 50.2% [27] on a susceptible variety (Daechang) in Korea. Groth et al. [30] is the only source with multi-year, multi-variety, and multi-susceptibility yield-loss data from blast inoculations.

The estimates put forth by Groth et al. [30] were used in this study to measure yield losses based on blast susceptibility ratings; these estimates were used because of the lack of locational and varietal-specific rates of yield response to blast. Furthermore, a static percentage yield loss would not be appropriate in this study because yield loss caused by blast is determined by the severity and timing of the infection; as such, a simulated range of yield losses was developed based on susceptibility rankings and empirically observed losses, as reported by Groth et al. [30]. For each susceptibility ranking, the study simulated the yield loss rate 1000 times, assuming a normal distribution such that the mean, standard deviation, minimum, and maximum of the simulations were equal to those reported by Groth et al. [30], as reported in Table 1. The mean percentage yield loss for the various blast susceptibility ratings are: 0.00%, 9.79%, 12.84%, 15.89%, and 18.32% for resistant, moderately resistant, moderately susceptible, susceptible, and very susceptible, respectively (Table 1).

**Table 1:** Simulated Blast Infection Rate and Yield Loss Rate by Blast Susceptibility Rating.

	Mean	Stdv	Max	Min
Infection rate (%) ^a^	21.52	12.01	46.95	0.00
Blast yield loss rate by susceptibility rating (%) ^b^				
Resistant	0.00	0.00	0.00	0.00
Moderately resistant	9.79	5.59	21.37	0.00
Moderately susceptible	12.84	3.85	22.88	0.00
Susceptible	15.89	5.35	24.53	0.00
Very susceptible	18.32	8.06	34.43	0.00

^a^Simulated using estimates of the yearly percentage of rice area that required a fungicide application, reported by Norman and Moldenhauer [23].

^b^Groth et al. [30].

### Cost of Blast Mitigation

The two most commonly used fungicides in the US to combat rice blast are Quilt Xcel^TM^ (active ingredients: 13.5% Azoxystrobin and 11.7% Propiconazole) and Quadris^TM^ (active ingredient: 22.9% Azoxystrobin), both of which are produced by Syngenta. Their prices vary by retailer and region and are affected by dealer rebates. In 2015, the average cost for Quilt Xcel to the grower was approximately $46.23 liter^-1^ in the Arkansas Delta. Comparatively, the average cost for Quadris was approximately US $72.65 liter^-1^ in the Arkansas Delta region. The recommended rate for Quilt Xcel application in the Mid-South is 1.28 l ha^-1^ and 0.73 l ha^-1^ for Quadris. Thus, the estimated cost of Quadris is US $53.08 ha^-1^ and US $59.11 ha^-1^for Quilt Xcel. Additionally, four crop dusting services in the Delta of Arkansas and Mississippi were contacted in August, 2015 to obtain an average application rate of aerial fungicide, which was recorded at $19.77 ha^-1^. The data was not available for the percentage of hectares treated with Quadris or Quilt Xcel; as such, the average price ($56.10 ha^-1^) of both fungicides (Quadris and Quilt Xcel) was taken and then added to the cost of aerial application for a total cost of $75.87 ha^-1^. It should be noted that both Quadris and Quilt Xcel are used to mitigate other common fungal diseases such sheath blight (*Rhizoctonia solani*) in the Mid-South. It is not uncommon to have simultaneous sheath blight and blasts infections. Table 1 and this study assume that if any fungicide is applied it is used to treat blast and all costs associated with application are attributed to blast outbreaks. Finally, because both the historical costs of aerial application and the costs of fungicide were prohibitive to obtain, they were assumed as constant across time, although adjusted for inflation.

### Blast Outbreak Scenario One

In the first scenario, all hectares of rice (*A*) produced in year *t* in county/parish *l* that were sown to non-resistant variety *i*—thus those not classified as resistant—were assumed to be treated with one application of fungicide. Scenario one was modeled as follows:

$${TC}_{t}^{1}=C_{h}\sum_{i}A_{ilt}$$ (1)

Where the annual total opportunity cost of blast for scenario one (${TC}_{t}^{1}$) is the summation of all actual historic hectares of susceptible rice varieties sown in each rice-producing county/parish in a given year, multiplied by the cost of the fungicide application per hectare (*C*_*h*_). The probability of a blast outbreak on 100% of the susceptible acres is negligible, but many producers apply a preventative fungicide application regardless of the presence of blast. One application of fungicide (Quadris) was built into the 2015 extension budgets of Arkansas, Louisiana, and Mississippi if a variety was blast susceptible [31, 32, 33]. It was assumed that once the fungicide was applied, there was no associated infection or yield loss from blast.

### Blast Outbreak Scenario Two

In the second scenario, the model simulated the area of susceptible varieties that were infected, at an average rate of 22.11%, as denoted in Table 1 [23]. The infected area was then treated with two applications of fungicide to mitigate this outbreak with no associated yield loss. Current production practices suggest two applications when blast is observed during the vegetative stage: one at the late booting stage and one seven days after the 90% panicle emergence of the main tiller when blast is spotted in a field [34]. Thus, scenario two was modeled as follows:

$${TC}_{t}^{2}=2C_{h}\lambda \sum_{i}A_{ilt}$$ (2)

Where the annual total opportunity cost of blast for scenario two (${TC}_{t}^{2}$) is the summation of all actual historic hectares of susceptible rice varieties (*i*) sown in each rice-producing county/parish (*l*) in a given year (*t*), multiplied by the simulated infection rate of blast (λ), and twice the cost of fungicide application per hectare (*C*_*h*_). Eq (2) is a function of time, given that the county/parish level varietal distribution and the area sown to susceptible varieties changes yearly. In this scenario, varietal blast ratings did not affect the probability of infection (yield loss will be a function of these ratings in scenario three); all varieties that were non-resistant had equal probabilities of infection.

### Blast Outbreak Scenario Three

In the third scenario, the model simulated a corresponding yield loss associated with the infections simulated in scenario two, based on the empirical yield-loss studies compiled by Groth et al. [30]. In this scenario, as in scenario two, the infected areas were assumed to be associated with two applications of fungicide; but unlike in scenario two, there was a subsequent yield loss associated with the infection. While a percentage yield loss was simulated for each susceptible variety, it was recognized that each variety had a different blast susceptibility rating and a different yield potential. As such, each variety’s average yield was denoted by county, as reported by each state’s extension service [17, 18, 19]. Scenario three was modeled as follows:

$${TC}_{t}^{3}={TC}_{t}^{2}+\sum_{i}\gamma_{i}P_{gt}Y_{il}$$ (3)

Where the annual total opportunity cost of blast for scenario three (${TC}_{t}^{3}$) is the summation of the annual total opportunity cost simulated for scenario two (${TC}_{t}^{2}$), and the product of the simulated yield loss due to blast (*γ*_*i*_*Y*_*il*_), associated with variety *i* and the season-average farm price for rice (*P*_*gt*_), relevant to variety *i*. The price *(P*_*gt*_) was measured in $ Mg^-1^ and aggregated at the grain-type level (*g* = 0 for medium, *g* = 1 for long grain), as reported by USDA [35]. The variable *γ*_*i*_ is the simulated percentage of the specific blast-rating yield loss shown in Table 1, and Y_*il*_ is the average yield for variety *i* in county/parish *l*.

### Impact on the U.S. Rice Market

Following the three scenarios, the RiceFlow model [36] was used to assess the impact of rice blast on the U.S. rice market according to the findings reported for each scenario. RiceFlow is a spatial supply-chain model of the global rice economy, and it is used extensively to assess different aspects of the global rice economy [11] [37] [38]. The RiceFlow model was calibrated to the market conditions for the calendar year of 2013. All monetary values are included in this paper and converted to 2014 USD, using annual consumer price index (CPI) retrieved from IMF [39]. In the model, the global rice economy was disaggregated into 73 regional markets and nine rice commodities derived from a combination of rice type (long, medium, and fragrant rice) and milling degree (paddy, brown, and milled rice), which allowed for the analysis of the impact of blast on the prices consumers pay in local markets.

In this study, a completely fixed supply of land and limited mobility of land across rice types in all countries was assumed. This assumption, coupled with the Leontief technology assumptions at each level of the production tree, produces results in very inelastic output supply functions. Hence, the results presented in this study can be understood as short-run outcomes controlling for potential supply-expansionary effects in other countries induced by rice blast outbreak in the US. The most up-to-date calibrated version of the RiceFlow model is for the 2013 production year; therefore, all results elicited from it will only be from 2013 since the model is not calibrated for each production year. From a consumer demand standpoint, we assume that all genetic properties (texture, aroma, palatability, amylose content, cooking time, etc.) remain the same besides the addition of a blast resistant gene. As such, consumer demand should not be altered between rice varieties with the addition of blast resistance given the fact that all else is assumed to be held equal.

### The Environmental Impacts of Blast Resistance

A lifecycle assessment (LCA) was performed to provide a quantitative comparison of the cradle-to-farm gate environmental benefits of rice blast elimination in the Mid-South of the United States. The goal was to provide a comparison (rice production with and without the presence of blast) for the functional unit of 1 kg of rice that is dried to 12.5% moisture at the farm-gate ready for transportation to milling. The principal differences between the two scenarios are yield and fungicide application. Blast-susceptible varieties subject to infection rates and yield losses are described on Table 1 and were subsequently sprayed with fungicide. The inputs for each system in terms of planting, fertilizer and pesticide application (except as noted), and harvesting with the same crop area were taken from the University of Arkansas extension budgets [6] and used in the LCA. The TRACI 2.1 LCA framework, which was developed by the US Environmental Protection Agency for conditions in the United States [40], was used to estimate potential environmental impacts arising from differences in production. Average yield loss and the probabilities of blast infections were taken from Table 1 and were incorporated into the LCA. In order to minimize bias in the comparison between the two scenarios, a paired Monte Carlo simulation approach was adopted using SimaPro 8.1, which selects variates from each unit process in the supply chain and computes the difference between the two (blast susceptible vs. resistance) production systems. This approach ensures that additional variability from independent simulations of the supply chains is not introduced. From this methodology, the differences were ascertained between the presence of blast in fields and blast-resistant rice production from a holistic environmental standpoint.

## Results

The total (aggregated annual) economic cost results of scenarios one, two, and three are presented in Tables 2 and 3, and the results for the U.S. rice market effect are presented in Table 4. For state-level results, see S2, S3, S4 and S5 Tables. S1 File provides all pertinent data used in the calculation of the results. The results from scenario one, where all hectares of rice production planted to non-resistant varieties were sprayed once with fungicide, indicate on average that $42.84 million is spent annually in Arkansas, Louisiana, and Mississippi on blast prevention. In scenario two, where a simulated percentage of historical plantings to blast-susceptible varieties were infected, it was estimated that Mid-South producers spend an average of $18.43 million on blast mitigation, given an assumed average infection rate of 21.52%, and that two aerial applications of fungicide were applied at a cost of $151.73 ha^-1^. If the maximum assumed infection rate of 46.95% is applied to the susceptible hectares, then the potential economic loss would reach $40.25 million annually. Lastly, the results from scenario three indicate that an average of $50.91 million is lost due to yield loss in addition to the $18.43 million spent on mitigation. Thus, on average, a total of $69.34 million is lost annually to blast. If the maximum infection rate of 46.95% is applied to the susceptible hectares, then the potential economic loss—mitigation ($40.20 million) plus yield loss ($163.28 million)—is estimated to be $203.49 million annually. The calculated potential economic loss as a share of the total value of rice production in each state for the period of 2002–2014 is estimated at 3.98%, 4.02%, and 4.92% for Mississippi, Louisiana, and Arkansas, respectively. The total value of rice production was retrieved from USDA [35] and converted to 2014 dollars using annual CPI retrieved from IMF [39]. Overall, the potential economic loss for scenario three is estimated at 4.08% as a share of the total value of rice production in the Mid-South of the US.

**Table 2:** Total Economic Cost of Blast Prevention and Simulated Mitigation by Fungicide Application to All Susceptible Rice Hectares in the Mid-South: 2002–2014.

Year	Scenario one		Scenario two			
	Rice area susceptible to blast (ha)^a^	Prevention cost for blast susceptible area ($)^b^^c^	Rice area infected with blast (ha)^d^		Mitigation cost for blast infected area ($)^b^^c^	
			Mean	Max	Mean	Max
2002	843,692	48,645,076	181,532	396,395	20,933,343	45,710,203
2003	814,340	48,188,321	175,217	382,604	20,736,789	45,281,005
2004	873,534	53,016,510	187,953	410,416	22,814,494	49,817,898
2005	883,399	54,955,645	190,076	415,051	23,648,958	51,640,040
2006	690,733	44,542,067	148,621	324,530	19,167,703	41,854,738
2007	635,952	42,456,886	136,834	298,792	18,270,391	39,895,361
2008	618,693	42,712,820	133,121	290,683	18,380,527	40,135,854
2009	705,768	48,724,207	151,856	331,594	20,967,395	45,784,560
2010	729,760	50,934,193	157,018	342,866	21,918,415	47,861,212
2011	400,957	28,897,689	86,272	188,383	12,435,488	27,154,222
2012	359,502	26,455,403	77,352	168,906	11,384,504	24,859,286
2013	400,389	29,767,986	86,149	188,116	12,810,001	27,972,012
2014	495,752	37,610,232	106,668	232,921	16,184,740	35,341,117
Avg.	650,190	42,839,003	139,898	305,481	18,434,827	40,254,424
Total	8,452,470	556,907,033	1,818,668	3,971,256	239,652,747	523,307,508

^a^ Annual varietal area planted to blast susceptible varieties in Arkansas, Louisiana and Mississippi (Proceedings of the Rice Technical Working Group (RTWG) [4].

^b^ Values in 2014 $; deflated with consumer price index retrieved from IMF [39].

^c^ Fungicide application at a rate of 1.01 l ha^-1^ and at a cost $ 75.87 ha^-^1 ($19.77 ha-1 for areal application and $51.10 ha-1 for fungicide).

^d^ Simulated using infection rates as shown on Table 1 and blast susceptible hectares.

Scenario one: All susceptible hectares sprayed once with fungicide to prevent blast outbreak. Scenario two: Simulated blast outbreak (Table 1) on susceptible hectares are sprayed twice with no associated yield loss. See S2 and S3 Tables for state specific results.

**Table 3:** Total Economic Cost of Blast Mitigation by Applying Two Application of Fungicide to Simulated Blast Infected Hectares in the Mid-South with Yield loss: 2002–2014.

Year	USA Season-average rice price ($ Mg^-1^) ^a^		Average total yield loss on blast infected area ($) ^a^		Average total loss on blast infected area ($) ^b^	
	Medium grain ^c^	Long grain	Mean	Max	Mean	Max
2002	171.19	120.41	28,061,174	86,794,710	48,994,517	132,446,185
2003	282.01	215.62	53,478,812	168,289,542	74,215,601	213,512,371
2004	201.43	202.82	45,979,853	146,330,860	68,794,348	196,084,753
2005	253.62	195.09	54,674,973	170,817,453	78,323,931	222,391,147
2006	313.26	245.17	51,567,061	162,691,215	70,734,764	204,492,179
2007	367.51	312.13	58,890,062	185,479,211	77,160,453	225,323,315
2008	441.19	401.19	66,029,792	207,399,857	84,410,319	247,484,145
2009	381.94	324.12	66,761,759	216,361,637	87,729,154	262,087,374
2010	359.02	280.00	67,678,652	225,152,490	89,597,067	272,952,211
2011	331.79	287.71	36,620,071	122,085,370	49,055,558	149,204,705
2012	334.16	311.95	36,515,535	120,599,034	47,900,038	145,426,381
2013	346.89	337.44	40,446,988	132,249,474	53,256,989	160,185,549
2014	341.38	315.94	55,088,962	178,442,700	71,273,701	213,738,412
Avg.	317.34	273.05	50,907,207	163,284,119	69,342,034	203,486,825
Total			661,793,693	2,122,693,551	901,446,439	2,645,328,727

^a^ Values in 2014 $; deflated with consumer price index retrieved from IMF [39].

^b^ Calculated as the summation of mitigation costs of the blast infected area presented on Table 2 and the average total yield loss on blast infected area from Table 3.

^c^ USDA reports medium grain prices from 2002–2008 as USA average and prices and reports 2009–2014 prices as Mid-South (Arkansas, Louisiana, Mississippi, Missouri, and Texas) averages. Price data retrieved from USDA [35].

All blast susceptible hectares are infected with the simulated blast rate on Table 1 and then subsequently sprayed twice with fungicide and an associated yield loss occurs dependent on the blast resistance rate presented on Table 1. See S4 Table for state specific results

**Table 4:** Impact of Simulated Blast Infected Hectares in the Mid-South with Yield loss on Selected U.S. Rice Market Variables in 2013.

Variables	All Rice			Long Grain Rice			Medium Grain Rice		
	Base^a^	Counter^b^	Change	Base^a^	Counter^b^	Change	Base^a^	Counter^b^	Change
	1000 Mg, paddy basis			1000 Mg, paddy basis			1000 Mg, paddy basis		
Production paddy rice	9,051	9,148	97	6,245	6,330	85	2,806	2,818	12
Change stock	-147	-147	0	-101	-101	0	-46	-46	0
Export paddy rice	1,520	1,542	22	1,520	1,542	22	0	0	0
Domestic sales paddy rice	7,678	7,753	75	4,826	4,889	63	2,852	2,864	12
Export brown rice	341	345	4	65	66	1	276	279	3
Import brown rice	14	14	0	14	14	0	0	0	0
Domestic sales brown rice	7,351	7,422	71	4,775	4,837	62	2,576	2,585	10
Export milled rice	2,774	2,836	61	1,770	1,823	53	1,004	1,013	9
Import milled rice	856	846	-10	124	120	-4	1	1	0
Domestic demand milled rice^c^	5,432	5,432	0	3,129	3,134	5	1,573	1,574	1
Exports	4,636	4,723	87	3,355	3,431	76	1,281	1,292	11
Imports	869	859	-10	138	134	-4	1	1	0
Paddy farm gate ($ Mg^-1^)^d^	351	348	-2	337	335	-3	380	379	-1
Milled rice retail ($ Mg^-1^)^d^	2,397	2,385	-12	2,134	2,120	-14	2,683	2,674	-9
Farm gate production ($ million)^d^	3,173	3,187	14	2,107	2,120	13	1,066	1,067	1
Retail consumption ($ million)^c^^,^^d^	9,116	9,075	-42	4,677	4,646	-32	2,953	2,943	-10

^a^ Simulates the domestic rice market as if all the cost increases and yield losses estimated in the scenario three were present.

^b^ Simulates the domestic rice market as if all the cost increases and yield losses estimated in the scenario three were eliminated.

^c^ For all rice, it includes 730 Mg^-1^ of fragrant rice imported in the benchmark.

^d^ Values in 2014 $; deflated with consumer price index retrieved from IMF [39].

Furthermore, the potential economic loss is also increased by the proportion of rice planted to varieties that are relatively less resistant (moderately susceptible, susceptible, or very susceptible) to blast. For our study’s timeframe (2002–2014), the proportion of rice planted to varieties that are susceptible to blast are estimated at 97.83%, 88.62%, and 81.73% in Mississippi, Louisiana, and Arkansas, respectively.

### Consumer Impacts of Rice Blast

The RiceFlow model was used to present the counterfactual case of blast alleviation and its effect on consumer prices. In other words, the model elicited the changes in the domestic rice market as if all the cost increases and yield losses estimated in the scenarios above were eliminated. Table 4 presents the results for the key selected variables from the estimated counterfactual decreased costs and increased yields associated with blast from the scenarios. The cost savings due to lower pesticide use (scenarios 1 and 2) are small, relative to the total production cost. Consequently, the cost savings generate no significant changes in the U.S. rice supply chain; however, U.S. consumers are expected to save an estimated $42 million when the yield losses and mitigation costs are accounted for (scenario 3). This result is exclusively due to lower rice prices. Most of the benefits ($32 million) exist in the consumption of long-grain rice, which undergoes the largest decrease in prices. Thus, it appears that blast alleviation does increase rice yields and production, as well as subsequently lower rice prices for long-grain rice. In turn, blast alleviation improves the competitiveness of U.S. rice and expands long-grain exports by 76,000 Mg or 2.3% and all rice exports by 87,000 Mg or 1.9%. In other words, the results suggest that the excess supply generated by the alleviation of rice blast in the US could be sufficient to feed a million people every year at the average per-capita consumption of 65 Kg. This suggestion is impressive considering the US is a small rice producer by global standards and likely experiences less loss from blast than the global average because of its ongoing investment in production technology and management.

## Environmental Impacts of Blast

We evaluated the environmental impacts associated with blast infection in rice through a counterfactual argument. Specifically, we presented comparisons of the current average condition in which a susceptible acreage is compared to a resistant acreage. The difference between the two is manifested in yield loss, whose probability is derived from Table 1. The ranges for infection rate in yield loss of a susceptible variety are compared in Table 5. The susceptible rating was chosen to analyze from all possibilities of susceptible ratings (moderately resistant, moderately susceptible, susceptible and very susceptible) because, in 2014, it accounted for the highest amount of acreage sown in the Mid-South, 29.14% [4]. We followed a cradle-to-farm gate LCA approach in performing this comparison, meaning that all inputs from production of fertilizers, through cultivation, harvest, and drying to a moisture content of 12%, have been included. The only differences between the two scenarios were yield differences and reduction in application of crop protection chemicals. Because these differences are uni-directional, and no other differences between the scenarios were introduced, the Monte Carlo simulations did not result in uncertainty regarding whether or not there are benefits from the elimination of blast. Notably, because there is uncertainty inherent in the system model, there is also uncertainty in the mean values for both scenarios, but no uncertainty that they are significantly different. The students t-tests show that for a pairwise comparison of 1000 simulations, the P value is less than 10^−8^ (Table 5).

**Table 5:** Results of the Categories in the Life Cycle Analysis Comparison of Blast-resistant Rice Production vs. Blast-susceptible Rice Production, based on 1000 Monte Carlo Simulations.

TRACI Impact category	Units	Description	Resistant	Susceptible^a^^b^	
Acidification	kg SO_2_ eq	Terrestrial acidification driven by acid gases	6.79E-03	7.03E-03	p<0.0001^c^
Carcinogens	CTUh	Human toxicity units	1.09E-07	1.13E-07	p<0.0001
Ecotoxicity	CTUe	Ecosystems toxicity units	3.69E+01	3.82E+01	p<0.0001
Eutrophication	kg N eq	Freshwater and marine eutrophication driven by nutrient runoff	5.32E-03	5.51E-03	p<0.0001
Fossil fuel depletion	MJ surplus	Nonrenewable energy consumption	1.03E+00	1.06E+00	p<0.0001
GWP	kg CO_2_ eq	Accumulated greenhouse gas emissions (IPCC 2006 characterization factors)	1.56E+00	1.61E+00	p<0.0001
Non-carcinogens	CTUh	Human toxicity units	-1.57E-07	-1.63E-07	p<0.0001
Ozone depletion	kg CFC-11	Accumulated ozone-depleting compounds emissions	1.13E-07	1.17E-07	p<0.0001
Respiratory effects	kg PM2.5 eq	Primary and secondary particulate emissions	5.24E-04	5.43E-04	p<0.0001
Smog	kg O_3_ eq	Small forming potential	6.24E-02	6.46E-02	p<0.0001

^a^ Yield loss (kg/ha) and probabilities associated with susceptible rice production are derived from Table 1.

^b^ All inputs are assumed to be identical with the exception of one application of Quilt XcelTM (13.5% Azoxystrobin and 11.7% Propiconazole) at a rate of 1.28 liters per hectare and one application of QuadrisTM (22.9% Azoxystrobin) at a rate of 0.73 liters per hectare for blast-infected varieties with probabilities given on Table 1.

^c^ Method: TRACI 2.1 V1.03 / US 2008, confidence interval: 95.

These simulations show that blast resistance in rice production results in lower global warming potential (GWP), carcinogenicity, ecotoxicity, eutrophication, fossil fuel depletion, and smog and ozone depletion (Table 5). The negative results for non-carcinogens are the result of modeling heavy metal uptake by the rice plants-the higher the yield, the greater the uptake, which results in decreased ecotoxicity at the farm; however, there is high uncertainty in these results, and we do not recommend any mitigation actions be taken on the basis of this result. Importantly, in using the well-established categories defined by the TRACI 2.1 LCA framework, it is evident from Table 5 that blast resistance in rice leads to multiple environmental improvements, as compared to blast-susceptible varietal production. While these results are intuitive from a comparative statistics perspective, higher yields and less inputs should have less of an environmental impact quantifying the environmental benefits of blast resistance demonstrates the value of embedded seed technology, which has previously mentioned is frequently overlooked or discounted.

## Conclusions

The results from this study indicate that blast alleviation from even a relatively small global rice producer like the US can have far-reaching implications for rice producers and consumers. Researchers suggest that crop production will need to double (2.4% annual increase) by the year 2050 to meet the rising global demand [41]; however, an analysis of crop-yield trends shows that these needs will not be met at the current rate of increase [41, 42]. As the rice yield gap closes and the yield ceiling approaches, maintenance breeding for pathogen resistance is one way to increase food supply without increasing yield potential. Notably, economists and policy-makers tend to undervalue the opportunity cost of this type of informative agricultural research, specifically with regard to maintenance breeding. In light of this, our study highlights the importance of maintenance breeding in terms of increasing food security as well as reducing environmental degradation. Importantly, both of these attributes are often forgotten in the cost-benefit analysis of agricultural research and development. While the most tangible outcome of a breeding program of any type is increased yield; this study has shown that not accounting for the benefits of biotic stress-resistance can substantially reduce the overall benefits.

While conventional breeding can produce blast-resistant varieties, the majority of blast- resistant rice in the US is hybridized. With this in mind, cisgenic rice breeding could also be used as a type of maintenance breeding technique to simultaneously “maintain” high yields and breed more quickly than conventional breeding for pathogen resistance to diseases like blast. Furthermore, there are no commercially bred cisgenic rice lines available, given that cisgenic breeding falls under the GM umbrella. While cisgenics is not a permanent solution for blast resistance (the pathogen will eventually evolve and a new resistance gene will be required), it can speed up the dissemination of a resistance gene. Accordingly, this study illuminates some of the potential benefits of cisgenic rice adoption in light of the complexity of global GM acceptance. While blast alleviation alone will not be the catalyst for GM adoption, it is one piece of the puzzle in helping policy-makers and consumers make better-informed decisions about GM adoption and the importance of funding research for blast alleviation from both a food security and environmental standpoint.

## Supporting Information

S1 Table Summary Statistics of Varieties by State. (PDF)

S2 Table Total Economic Cost of Blast Prevention by Applying One Application of Fungicide to All Susceptible Rice Hectares by State: 2002–2014. (PDF)

S3 Table : Total Economic Cost of Blast Mitigation by Applying Two Applications of Fungicide to Simulated Blast-Infected Rice Hectares by State with No Yield Loss: 2002–2014. (PDF)

S4 Table Total Economic Cost of Blast Mitigation by Applying Two Applications of Fungicide to Simulated Blast-Infected Rice Hectares by State with Yield Loss: 2002–2014. (PDF)

S5 Table Varieties Associated with the Highest Annual County Economic Cost of Blast Mitigation by Applying Two Applications of Fungicide to Simulated Blast-Infected Rice Hectares with Yield Loss: 2002–2014. (PDF)

S1 File Data Used in Estimation (CSV)

## References

[1] FAO. FAO’s director-general on how to feed the world in 2050. Insights from an Expert Meeting at FAO. 2009; 1: 1–35.

[2] Skamnioti P, Gurr SJ. Against the grain: safeguarding rice from rice blast disease. Trends Biotechnol. 2009; 27(3): 141–50. 10.1016/j.tibtech.2008.12.002 19187990

[3] Wang G-L, Valent B. Advances in genetics, genomics and control of rice blast disease Springer Science+Business Media B.V. 2009.

[4] Rice Technical Working Group (RTWG). Rice Acreage Summaries. In: Proceedings of the Rice Technical Working Group (RTWG) [CDROM]; various years 2001–2013.

[5] Khush GS, Jena KK. Current Status and Future Prospects for Research on Blast Resistance in Rice (Oryza sativa L.) In: Wang G-L, Valent B, editors. Advances in genetics, genomics and control of rice blast disease. Springer Netherlands; 2009 pp. 1–10.

[6] University of Arkansas Cooperative Extension Service (UACES). 2016 crop enterprise budgets for Arkansas field crops planted in 2016 UACES 2016 Avalable: http://uaex.edu/farm-ranch/economics-marketing/farm-planning/budgets/docs/budgets2016/Manuscript_2016.pdf

[7] Zhou E, Jia Y, Singh P, Correll J, Lee F. Instability of the Magnaporthe oryzae avirulence gene AVR-Pita alters virulence. Fungal Genet. Biol. 2007; 44: 1024–1034. 10.1016/j.fgb.2007.02.003 17387027

[8] Wang X, Jia M, Ghai P, Lee F, Jia Y. Genome-wide association of rice blast disease resistnace and yield-related componets of rice. Molecular Plant-Microbe Interactions. 2015; 28: 1383–1392. 10.1094/MPMI-06-15-0131-R 26284908

[9] Marasas C N, Smale M, Singh RP. The economic impact of productivity maintenance research: Breeding for leaf rust resistance in modern wheat. Agricultural Economics. 2003; 29: 253–263.

[10] Peng S, Huang J, Cassman KG, Laza RC, Visperas RM, Khush GS. The importance of maintenance breeding: A case study of the first miracle rice variety-IR8. Field Crops Res. 2010; 119(2–3): 342–347.

[11] Qu S, Liu G, Zhou B, Bellizzi M, Zeng L, Dai L, et al The broad-spectrum blast resistance gene Pi9 encodes a nucleotide-binding site-leucine-rich repeat protein and is a member of a multigene family in rice. Genetics. 2006; 172(3): 1901–1914. 10.1534/genetics.105.044891 16387888 PMC1456263

[12] Schouten HJ, Krens FA, Jacobsen E. Cisgenic plants are similar to traditionally bred plants: international regulations for genetically modified organisms should be altered to exempt cisgenesis. EMBO Reports. 2006a; 7(8): 750–3. 10.1038/sj.embor.7400769 16880817 PMC1525145

[13] Andersson HC, Arpaia S, Bartsch D, Casacuberta J, Davies H, du Jardin P, et al Scientific opinion addressing the safety assessment of plants developed through cisgenesis and intragenesis. EFSA Journal. 2012; 10(2): 2561.

[14] Schouten HJ, Krens FA, Jacobsen E. Do cisgenic plants warrant less stringent oversight? Nature Biotechnol. 2006b; 24(7): 753. 16841052 10.1038/nbt0706-753

[15] Nalley LL, Moldenhauer KA, Lyman N. The genetic and economic impact of the University of Arkansas’s rice breeding program: 1983–2007. JAAE. 2011; 43(01): 131–142.

[16] Peter M. Rice Blast Research Reveals Details of How a Fungus Invades Plants. K-State Today. 19 Jun 2013. Available: http://www.k-state.edu/today/announcement.php?id=9034&category=research&referredBy=K-State Today Archive. Accessed 7 Mar 2016.

[17] Arkansas Agricultural Experiment Station (AAES). Arkansas rice performance trials (ARPT). 2002–2015. Available: http://arkansasvarietytesting.com/home/rice/

[18] Louisiana State University Agricultural Center (LSU AgCenter). Rice varieties and management tips. 2003, 2014, 2015, and 2016. Available: http://www.lsuagcenter.com/NR/rdonlyres/4F5F83A3-20FA-4391-813D-9BB69C54E99F/100040/pub2270RiceVarieties2015FINAL.pdf

[19] Mississippi Agricultural and Forester Experiment Station (MAFES). Mississippi Rice Variety Trials. 2002–2015. Available: http://www.mafes.msstate.edu/variety-trials/archive.asp#rice

[20] Brennan JP. Measuring the contribution of new varieties to increasing wheat yields. Review of Marketing and Agricultural Economics. 1984; 52(03). Available: http://ideas.repec.org/a/ags/remaae/12281.html

[21] Blanchard T, Gautreaux K, Groth D, Linscombe S. Blast Outbreak on Rice Worst Seen in Years. LSU Ag Center 4 1 2016 Available: http://www.lsuagcenter.com/portals/communications/news/news_archive/2012/july/radio_tv/blast-outbreak-on-rice-is-worst-seen-in-years. Accessed 7 Mar 2016

[22] Groth D, Dischler C, Monte L, Frey M. Rice disease control studies LSU Ag Center 2014 Available: https://www.lsuagcenter.com/MCMS/RelatedFiles/{13E3E56A-0283-42BE-8CCF-2685728B9336}/2013-Final-Annual-Report.pdf. Accessed 31 Dec 2015.

[23] Norman R, Moldenhauer K, editors. B.R. Wells Arkansas Rice Research Studies. Arkansas Agricultural Experiment Station. Various years: 2002–2014. Available: http://arkansasagnews.uark.edu/1356.htm

[24] Bastiaans L, Kropff MJ. Effects of leaf blast on photosynthesis of rice 2. canopy photosynthesis. Neth. J. Plant Pathol. 1993; 99(4): 205–217.

[25] Ganesh NR, Gangadhara NB, Basavaraja NT, Krishna NR. Fungicidal management of leaf blast disease in rice. GJBB. 2012; 1(1): 18–21.

[26] Zeigler RS, Leong SA, Teeng PS. Rice Blast Disease. Commonwealth Agricultural Bureaux. Wallingford, UK: Commonwealth Agricultural Bureaux 1994.

[27] Bonman JM, Estrada BA, Kim CK, Lee EJ. Assessment of blast disease and yield loss in susceptible and partially resistant rice cultivars in two irrigated lowland environments. Plant Dis. 1991; 75(5): 462–466.

[28] Koutroubas SD, Katsantonis D, Ntanos DA, Lupotto E. Nitrogen utilisation efficiency and grain yield components of rice varieties grown under blast disease stress. Australas. Plant Path. 2008; 37(1): 53–59.

[29] Koutroubas SD, Katsantonis D, Ntanos DA, Lupotto E. Blast disease influence on agronomic and quality traits of rice varieties under Mediterranean conditions. Turk. J. Agric. 2009; 33(5): 487–494. http://journals.tubitak.gov.tr/agriculture/abstract.htm?id=10484

[30] Groth D, Dischler C, Monte L, Frey M. Rice disease control studies, 2014. In: The 105th Annual Research Report of the Rice Research Station. Crowley, Louisiana. Various years. Available: https://www.lsuagcenter.com/MCMS/RelatedFiles/%7B13E3E56A-0283-42BE-8CCF-2685728B9336%7D/2013-Final-Annual-Report.pdf. Accessed 31 Dec 2015.

[31] Falconer L, Allen T, Bond J, Golden B, Gore J, Pringle H. Mississippi rice planning budgets Mississippi State University Extension 2015 Available: http://msucares.com/pubs/publications/p2865.pdf. Accessed: 7 Mar 2016.

[32] Flanders A, Watkins B. Arkansas crop enterprise budgets U of A Division of Agriculture Research and Extension 2015 Available: http://uaex.edu/farm-ranch/economics-marketing/farm-planning/budgets/docs/budgets2016/Manuscript_2016.pdf. Accessed 7 Mar 2016.

[33] Salassi M,.Deliberto MA, Hilbun BM. Projected costs and returns crop enterprise budgets for rice production in Louisiana, 2015. 2015. Available: file:///C:/Users/ftsiboe/Downloads/2015RiceEnterpriseBudgets.pdf. Accessed: 7 Mar 2016.

[34] University of Arkansas Cooperative Extension Service (UACES). Rice production handbook U of A Division of Agriculture Research and Extension 2015 http://www.uaex.edu/publications/pdf/MP192/MP192.pdf. Accessed: 7 Mar 2016.

[35] USDA. National agricultural statistics service, quick stats and world agricultural supply and demand estimates report U.S. Department of Agriculture 2015.

[36] Durand-Morat A, Wailes EJ. Riceflow: a multi-region, multi-product, spatial partial equilibrium model of the world rice economy University of Arkansas, Department of Agricultural Economics and Agribusiness 2010 Available: http://econpapers.repec.org/RePEc:ags:uarksp:92010

[37] Briones RM, Durand-Morat A, Wailes EJ, Chavez EC. Climate change and price volatility: Can we count on the ASEAN plus three emergency rice reserve? Asian Development Bank 2012; (24).

[38] Wailes EJ, Durand-Morat A, Diagne M. Regional and national rice development strategies for food security in West Africa In: Schmitz A, Kennedy PL, Schmitz TG, editors. Food security in an uncertain world (Frontiers of Economics and Globalization, Volume 15). Emerald Group Publishing Limited; 2015 pp. 255–268.

[39] IMF. International Financial Statistics (IFS). International Monetary Fund 2015 Available: http://data.imf.org/?sk=5DABAFF2-C5AD-4D27-A175-1253419C02D1. Accessed 16 Oct 2015.

[40] Bare J, Gloria, Norris G. Development of the method and U.S. normalization database for life cycle impact assessment and sustainability metrics. Environmental Science & Technology. 2006; 40(16): 5108–5115. Available: http://www.ncbi.nlm.nih.gov/pubmed/16955915. Accessed 5 Feb 2016. 16955915 10.1021/es052494b

[41] Ray DK, Mueller ND, West PC, Foley JA. Yield trends are insufficient to double global crop production by 2050. PLoS ONE. 2013; 8(6): e66428 10.1371/journal.pone.0066428 23840465 PMC3686737

[42] Grassini P, Eskridge KM, Cassman KG. Distinguishing between yield advances and yield plateaus in historical crop production trends. Nat. Comm. 2013; 2918. 10.1038/ncomms3918 PMC3905725 24346131

