# Supplementary material for: Economic and Environmental Impact of Rice Blast Pathogen (Magnaporthe oryzae) Alleviation in the United States
Source: PLoS One. 2016 Dec 1;11(12):e0167295. doi: 10.1371/journal.pone.0167295 (PMC5131998; doi:10.1371/journal.pone.0167295)
Supplement: S1 Table — (PDF) [file pone.0167295.s001.pdf]

S1 Table. Summary Statistics of Varieties by State.

Variety	Number of observation	Blast Susceptibility	Highest State % of rice hectares			Yield (Mg.ha^{-1})		
			AR	LA	MS	AR	LA	MS
Ahrent	46	MR	2.55	-	-	8.44	-	-
Banks	23	R	3.27	-	-	10.58	-	-
Bengal	252	S	11.75	4.00	-	9.05	7.57	-
Bowman	7	S	-	-	2.43	-	-	9.94
Caffey	8	MR	-	1.01	-	-	9.19	-
Catahoula	46	R	-	5.08	-	-	7.31	-
Cheniere	385	S	11.54	24.35	18.26	8.79	7.84	10.41
CL111	245	S	6.66	33.78	12.45	8.76	8.29	11.90
CL121	47	S	1.39	2.23	-	8.01	6.32	-
CL131	144	MS	14.17	23.26	19.93	8.82	7.64	9.02
CL141	4	VS	-	0.14	-	-	5.36	-
CL142	30	S	8.53	0.14	-	7.87	7.87	-
CL151	348	VS	24.57	34.13	27.77	9.18	9.03	11.89
CL152	130	VS	8.63	3.92	17.06	8.53	8.29	11.57
CL161	373	S	20.43	31.46	18.47	8.80	7.72	8.27
CL171	86	S	20.10	12.19	20.56	7.91	7.56	8.88
CL261	58	VS	6.52	3.59	-	8.52	7.55	-
CL271	4	MR	-	0.15	-	-	9.17	-
CLXL4534	18	R	-	-	8.10	-	-	14.81
CLXL710	6	R	-	0.15	-	-	9.40	-
CLXL723	175	MR	11.15	2.69	7.21	10.29	11.47	12.07
CLXL729	329	MR	17.19	8.79	14.42	9.94	10.81	12.33
CLXL730	81	MR	5.25	3.23	0.73	10.55	10.48	10.35
CLXL745	359	MR	33.41	15.75	22.67	8.64	10.59	12.80
CLXL753	111	MR	12.84	5.83	4.50	12.18	11.07	14.26
CLXL8	148	R	5.97	1.86	2.93	10.43	9.05	9.41
Cocodrie	648	S	30.59	58.05	78.17	8.97	7.71	10.36
Cypress	169	S	4.09	31.97	2.12	7.61	7.28	7.28
Drew	23	R	4.61	-	0.08	9.09	-	9.09
Earl	2	MR	-	0.09	-	-	7.31	-
Francis	217	VS	17.26	0.91	2.06	9.72	7.28	10.11
Jazzman2	15	MR	-	8.94	-	-	6.41	-
Jefferson	27	S	-	1.35	1.93	-	6.42	6.42
Jupiter	291	S	15.25	14.19	-	10.09	8.53	-
Lagrue	29	S	3.57	-	-	10.02	-	-
Lemont	11	MR	-	-	5.88	-	-	7.83
Maybelle	9	VS	-	0.79	-	-	5.07	-
Mermentau	44	S	5.30	4.30	-	9.44	8.55	-
Neptune	23	R	-	1.64	-	-	7.22	-
Pirogue	13	MR	-	0.29	-	-	6.68	-
Priscilla	40	MS	-	-	16.67	-	-	9.36
Rex	50	VS	-	-	17.18	-	-	11.78
Roy J	75	S	15.47	-	-	10.86	-	-
Saber	6	R	-	0.45	0.10	-	6.73	8.02
Sabine	62	S	-	-	3.23	-	-	10.39
Trenasse	43	MS	-	10.40	-	-	8.20	-
Wells	473	S	47.51	7.76	6.06	9.51	7.92	9.63

Calculated by authors using annual data retrieved from the Proceedings of the Rice Technical Working Group [4].

[a]Locations in AR; Arkansas, Ashley, Chicot, Clay, Conway, Craighead, Crittenden, Cross, Desha, Drew, Faulkner, Greene, Independence, Jackson, Jefferson, Lafayette, Lawrence, Lee, Lincoln, Lonoke, Miller, Mississippi, Monroe, Perry, Phillips, Poinsett, Pope, Prairie, Pulaski, Randolph, St. Francis, White, and Woodruff.

[b]Locations in LA; Acadia, Allen, Avoyelles, Beauregard, Bossier, Caddo, Calcasieu, Caldwell, Cameron, Catahoula, Concordia, East Carroll, Evangeline, Franklin, Grant, Iberia, Iberville, Jefferson Davis, La Salle, Lafayette, Madison, Morehouse,

Natchitoches, Ouachita, Point Coupee, Rapides, Red River, Richland, St Mary, St. Landry, St. Martin, Tensas, Vermilion, West Baton Rouge, and West Carroll.

[c]Locations in MS; Adams, Bolivar, Coahoma, Desoto, Grenada, Holmes, Humphreys, Issaquena, Leflore, Panola, Quitman, Sharkey, Sunflower, Tallahatchie, Tate, Tunica, Washington, and Yazoo.
