# Supplementary material for: Economic and Environmental Impact of Rice Blast Pathogen (Magnaporthe oryzae) Alleviation in the United States
Source: PLoS One. 2016 Dec 1;11(12):e0167295. doi: 10.1371/journal.pone.0167295 (PMC5131998; doi:10.1371/journal.pone.0167295)
Supplement: S2 Table — (PDF) [file pone.0167295.s002.pdf]

S2 Table. Total Economic Cost of Blast Prevention by Applying One Application of Fungicide to All Susceptible Rice Hectares by State: 2002-2014.

Year	Rice area susceptible to blast (ha)[a]	Prevention cost for blast susceptible area ($)[b]	Blast prevention fungicide spraying (l) [c]
		Arkansas	
2002	525,765	30,314,232	528,394
2003	552,339	32,684,466	555,100
2004	563,487	34,199,166	566,305
2005	580,061	36,085,197	582,961
2006	489,450	31,562,326	491,898
2007	446,224	29,790,462	448,455
2008	384,862	26,569,807	386,787
2009	466,584	32,211,653	468,917
2010	465,384	32,481,823	467,711
2011	222,027	16,001,869	223,137
2012	191,309	14,078,251	192,266
2013	244,083	18,147,026	245,304
2014	322,470	24,464,205	324,083
		Louisiana	
2002	214,359	12,359,402	215,431
2003	178,563	10,566,426	179,456
2004	212,152	12,875,916	213,213
2005	202,613	12,604,408	203,626
2006	135,091	8,711,381	135,767
2007	123,536	8,247,416	124,154
2008	149,545	10,324,149	150,293
2009	140,229	9,681,008	140,930
2010	147,789	10,315,073	148,528
2011	117,658	8,479,818	118,246
2012	121,761	8,960,285	122,370
2013	111,402	8,282,480	111,959
2014	130,357	9,889,568	131,009
		Mississippi	
2002	103,568	5,971,441	104,085
2003	83,438	4,937,428	83,855
2004	97,895	5,941,429	98,384
2005	100,725	6,266,040	101,229
2006	66,191	4,268,359	66,522
2007	66,191	4,419,007	66,522
2008	84,286	5,818,864	84,707
2009	98,955	6,831,546	99,449
2010	116,587	8,137,297	117,170
2011	61,272	4,416,001	61,579
2012	46,432	3,416,868	46,664
2013	44,904	3,338,479	45,128
2014	42,924	3,256,458	43,139

[a] Annual varietal area planted to blast susceptible varieties in Arkansas, Louisiana and Mississippi from Proceedings of the Rice Technical Working Group [4].
[b] Values in 2014 $; deflated with consumer price index retrieved from IMF [39].
[c] Fungicide application at a rate of 1.01 l ha-1 and at a cost $ 75.87 ha-1 ($19.77 ha-1 for areal application and $51.10 ha-1 for fungicide)Scenario one: All susceptible hectares sprayed once with fungicide to prevent blast outbreak.
