# Supplementary material for: Economic and Environmental Impact of Rice Blast Pathogen (Magnaporthe oryzae) Alleviation in the United States
Source: PLoS One. 2016 Dec 1;11(12):e0167295. doi: 10.1371/journal.pone.0167295 (PMC5131998; doi:10.1371/journal.pone.0167295)
Supplement: S3 Table — (PDF) [file pone.0167295.s003.pdf]

S3 Table. Total Economic Cost of Blast Mitigation by Applying Two Applications of Fungicide to Simulated Blast-Infected Rice Hectares by State with No Yield Loss: 2002-2014.

Year	Rice area infected with blast (ha)[a]			Mitigation cost for blast infected area ($)[b]			Blast mitigation fungicide spraying (l) [c]		
	Min	Mean	Max	Min	Mean	Max	Min	Mean	Max
				Arkansas					
2002	211	113,126	247,022	24,300	13,045,066	28,485,303	426	228,514	498,985
2003	221	118,843	259,507	26,200	14,065,044	30,712,535	447	240,064	524,205
2004	226	121,242	264,745	27,414	14,716,862	32,135,849	456	244,909	534,786
2005	232	124,808	272,532	28,926	15,528,474	33,908,091	470	252,113	550,515
2006	196	105,312	229,960	25,300	13,582,156	29,658,096	396	212,730	464,520
2007	179	96,011	209,651	23,880	12,819,673	27,993,133	361	193,943	423,495
2008	154	82,809	180,821	21,298	11,433,735	24,966,788	312	167,273	365,259
2009	187	100,392	219,217	25,821	13,861,580	30,268,248	378	202,792	442,819
2010	187	100,134	218,653	26,037	13,977,841	30,522,118	377	202,270	441,679
2011	89	47,772	104,316	12,827	6,886,054	15,036,438	180	96,500	210,718
2012	77	41,163	89,883	11,285	6,058,267	13,228,877	155	83,149	181,565
2013	98	52,518	114,679	14,547	7,809,175	17,052,173	198	106,086	231,651
2014	129	69,384	151,507	19,610	10,527,635	22,988,222	261	140,156	306,045
				Louisiana					
2002	86	46,122	100,713	9,907	5,318,598	11,613,730	174	93,167	203,441
2003	72	38,420	83,895	8,470	4,547,030	9,928,929	145	77,609	169,468
2004	85	45,647	99,676	10,321	5,540,868	12,099,081	172	92,208	201,346
2005	81	43,595	95,194	10,104	5,424,031	11,843,954	164	88,062	192,293
2006	54	29,067	63,470	6,983	3,748,752	8,185,803	109	58,715	128,210
2007	50	26,581	58,041	6,611	3,549,095	7,749,830	100	53,693	117,244
2008	60	32,177	70,261	8,276	4,442,771	9,701,268	121	64,997	141,928
2009	56	30,172	65,884	7,760	4,166,010	9,096,929	114	60,948	133,086
2010	59	31,799	69,436	8,269	4,438,866	9,692,740	120	64,234	140,262
2011	47	25,316	55,280	6,797	3,649,104	7,968,211	95	51,138	111,665
2012	49	26,199	57,207	7,183	3,855,862	8,419,689	99	52,921	115,559
2013	45	23,970	52,340	6,639	3,564,184	7,782,778	90	48,419	105,728
2014	52	28,048	61,246	7,927	4,255,759	9,292,907	106	56,657	123,718
				Mississippi					
2002	42	22,284	48,660	4,787	2,569,679	5,611,170	84	45,014	98,292
2003	33	17,953	39,202	3,958	2,124,714	4,639,541	68	36,265	79,188
2004	39	21,063	45,994	4,763	2,556,764	5,582,968	79	42,548	92,908
2005	40	21,672	47,324	5,023	2,696,453	5,887,995	82	43,778	95,595
2006	27	14,242	31,099	3,422	1,836,795	4,010,839	54	28,769	62,820
2007	27	14,242	31,099	3,542	1,901,623	4,152,398	54	28,769	62,820
2008	34	18,135	39,600	4,664	2,504,021	5,467,798	68	36,633	79,993
2009	40	21,291	46,492	5,476	2,939,806	6,419,382	80	43,009	93,914
2010	47	25,085	54,777	6,523	3,501,708	7,646,355	94	50,672	110,649
2011	25	13,184	28,788	3,540	1,900,330	4,149,574	50	26,631	58,151
2012	19	9,990	21,815	2,739	1,470,374	3,210,720	38	20,181	44,067
2013	18	9,662	21,097	2,676	1,436,642	3,137,061	36	19,517	42,616
2014	17	9,236	20,167	2,610	1,401,345	3,059,988	35	18,656	40,738

[a] Simulated using infection rates as shown on Table 1 and blast susceptible hectares.
[b] Values in 2014 $; deflated with consumer price index retrieved from IMF [39].
c Fungicide application at a rate of 1.01 l ha-1 and at a cost $ 75.87 ha-1 ($19.77 ha-1 for areal application and $51.10 ha-1 for fungicide)Scenario one: All susceptible hectares sprayed once with fungicide to prevent blast outbreak.
Scenario two: Simulated blast outbreak (Table 1) on susceptible hectares are sprayed twice with no associated yield loss.
