# Supplementary material for: Economic and Environmental Impact of Rice Blast Pathogen (Magnaporthe oryzae) Alleviation in the United States
Source: PLoS One. 2016 Dec 1;11(12):e0167295. doi: 10.1371/journal.pone.0167295 (PMC5131998; doi:10.1371/journal.pone.0167295)
Supplement: S4 Table — (PDF) [file pone.0167295.s004.pdf]

S4 Table. Total Economic Cost of Blast Mitigation by Applying Two Applications of Fungicide to Simulated Blast-Infected Rice Hectares by State with Yield Loss: 2002-2014.

Year	Average total yield loss on blast infrected area ($) [a]			Average total loss on blast infected area ($) [ab]		
	Min	Mean	Max	Min	Mean	Max
	Arkansas					
2002	11,738	19,728,842	60,086,278	18,823	32,773,908	87,497,276
2003	25,797	40,900,588	126,240,147	33,436	54,965,633	155,794,378
2004	21,305	32,305,827	101,008,077	29,298	47,022,689	131,931,942
2005	27,143	40,990,040	127,323,840	35,576	56,518,514	159,953,108
2006	25,128	38,861,802	122,344,237	32,504	52,443,958	150,883,797
2007	28,554	44,768,310	140,922,698	35,516	57,587,983	167,860,088
2008	27,958	42,783,998	136,208,324	34,168	54,217,733	160,233,505
2009	28,266	42,959,528	140,162,472	35,794	56,821,107	169,289,173
2010	34,159	45,180,527	150,189,708	41,750	59,158,368	179,560,704
2011	14,958	20,474,405	66,670,623	18,698	27,360,459	81,139,972
2012	16,117	22,080,372	72,174,362	19,407	28,138,639	84,904,320
2013	17,953	26,385,139	85,772,327	22,195	34,194,314	102,181,389
2014	27,558	38,619,037	124,535,597	33,275	49,146,672	146,656,833
	Louisiana					
2002	2,698	5,233,595	16,602,267	5,587	10,552,193	27,389,253
2003	4,925	8,834,998	27,131,780	7,395	13,382,028	36,686,246
2004	4,833	9,015,432	27,799,692	7,843	14,556,301	39,442,464
2005	5,206	9,455,436	29,068,271	8,152	14,879,467	40,465,538
2006	4,353	8,411,044	26,585,898	6,389	12,159,796	34,039,528
2007	4,304	8,169,140	25,479,587	6,232	11,718,235	32,696,173
2008	7,365	13,408,448	41,232,296	9,777	17,851,220	50,567,687
2009	8,936	12,973,428	43,207,360	11,198	17,139,438	51,961,205
2010	7,589	10,298,908	35,025,112	10,000	14,737,774	44,352,296
2011	7,090	9,599,853	32,827,734	9,072	13,248,957	40,495,429
2012	6,240	9,261,877	30,878,169	8,334	13,117,739	38,980,315
2013	4,723	8,693,446	28,200,638	6,658	12,257,630	35,287,291
2014	6,107	10,971,312	35,312,128	8,418	15,227,071	43,773,838
	Mississippi					
2002	1,534	3,098,737	10,106,164	2,929	5,668,416	15,215,447
2003	1,243	3,743,226	14,917,616	2,397	5,867,940	19,181,004
2004	1,782	4,658,594	17,523,091	3,170	7,215,358	22,606,695
2005	1,951	4,229,497	14,425,342	3,416	6,925,950	19,786,690
2006	2,180	4,294,215	13,761,080	3,178	6,131,010	17,413,172
2007	3,022	5,952,613	19,076,926	4,054	7,854,236	22,857,916
2008	5,855	9,837,346	29,959,237	7,215	12,341,367	35,220,821
2009	6,425	10,828,803	32,991,804	8,021	13,768,609	39,169,083
2010	8,077	12,199,217	39,937,670	9,979	15,700,925	47,295,647
2011	3,722	6,545,813	22,587,012	4,755	8,446,142	26,365,431
2012	3,204	5,173,286	17,546,503	4,002	6,643,660	20,470,042
2013	3,287	5,368,404	18,276,509	4,067	6,805,045	21,132,978
2014	3,438	5,498,613	18,594,975	4,199	6,899,958	21,381,265

[a] Values in 2014 $; deflated with consumer price index retrieved from IMF [39].

[b] Calculated as the summation migration costs of blast infected area presented on Table A3 and the average total yield loss on blast infected area from Table A4.

All blast susceptible hectares are infected with the simulated blast rate on Table 1 and then subsequently sprayed twice with fungicide and an associated yield loss occurs dependent on the blast resistance rate presented on Table 1.
