# Supplementary material for: Economic and Environmental Impact of Rice Blast Pathogen (Magnaporthe oryzae) Alleviation in the United States
Source: PLoS One. 2016 Dec 1;11(12):e0167295. doi: 10.1371/journal.pone.0167295 (PMC5131998; doi:10.1371/journal.pone.0167295)
Supplement: S5 Table — (PDF) [file pone.0167295.s005.pdf]

S5 Table. Varieties Associated with the Highest Annual County Economic Cost of Blast Mitigation by Applying Two Applications of Fungicide to Simulated Blast-Infected Rice Hectares with Yield Loss: 2002-2014.

Year	Variety	Total loss on blast infected area ($) [ab]		
		Avg.	Max	Min
		**Arkansas**		
2002	Bengal	243,249	1,691,653	104
2003	Wells	915,286	2,396,181	42,545
2004	Wells	718,730	2,008,518	20,812
2005	Wells	880,603	2,439,187	21,545
2006	Wells	613,469	2,194,291	9,231
2007	Wells	829,623	2,727,067	35,025
2008	Francis	520,813	2,226,653	11,065
2009	Francis	416,693	2,001,609	9,941
2010	Cl151	815,925	2,825,515	24,499
2011	Jupiter	316,136	1,647,039	7,773
2012	Cl151	419,405	1,555,250	30,486
2013	Jupiter	269,252	1,763,021	9,407
2014	Jupiter	494,270	2,995,353	8,564
		**Louisiana**		
2002	Cocodrie	184,168	840,409	171
2003	Cocodrie	262,134	1,080,865	6,471
2004	Cocodrie	226,164	1,153,891	1,280
2005	Cl161	217,526	1,424,089	2,334
2006	Cheniere	138,002	639,611	1,105
2007	Cocodrie	237,653	996,393	888
2008	Cl161	315,932	1,799,059	1,531
2009	Cl151	306,768	1,610,771	6,262
2010	Cl151	341,158	1,763,376	5,921
2011	Cl151	341,862	1,598,445	6,383
2012	Cl151	234,819	1,365,472	2,812
2013	Cl111	309,151	1,269,236	6,509
2014	Cl111	329,616	1,503,253	2,510
		**Mississippi**		
2002	Cocodrie	314,993	1,264,734	6,605
2003	Cocodrie	364,489	1,286,684	8,361
2004	Cocodrie	297,379	1,288,841	5,717
2005	Cocodrie	216,594	809,899	12,581
2006	Cocodrie	378,745	1,405,335	33,106
2007	Cocodrie	498,071	1,674,728	37,060
2008	Cocodrie	568,752	1,632,170	4,240
2009	Cl151	388,656	1,982,035	6,236
2010	Cl151	396,115	1,762,860	6,406
2011	Cl151	127,558	792,534	7,074
2012	Rex	75,570	377,465	1,523
2013	Rex	82,711	360,883	1,294
2014	Rex	86,752	381,348	1,368

[a] Estimated as the value of yield loss and fungicide application after all hectares planted to variety are infected with the simulated blast rate on Table 1 and then subsequently sprayed twice with fungicide and an associated yield loss occurs dependent on the blast resistance rate presented on Table 1.
[b] Values in 2014 $; deflated with consumer price index retrieved from IMF [39].
